# Intravenous thrombolysis upon flow restoration improves outcome in endovascular thrombectomy

**DOI:** 10.1136/jnis-2022-019522

**Published:** 2022-10-28

**Authors:** Johannes M Weller, Franziska Dorn, Gabor C Petzold, Felix J Bode, GSR-ET investigators, A Alegiani

**Affiliations:** 1 Division of Vascular Neurology, Department of Neurology, University Hospital Bonn, Bonn, Germany; 2 Department of Neuroradiology, University Hospital Bonn, Bonn, Germany; 3 Vascular Neurology Research Group, German Center for Neurodegenerative Diseases (DZNE), Bonn, Nordrhein-Westfalen, Germany

**Keywords:** Stroke, Thrombectomy, Thrombolysis

## Abstract

**Background:**

We hypothesized that ongoing IV thrombolysis (IVT) at flow restoration in patients with acute ischemic stroke (AIS) treated with IVT and endovascular thrombectomy (ET) is associated with improved outcome.

**Methods:**

We included patients with IVT and successful recanalization (modified Thrombolysis in Cerebral Infarction score ≥2b) after ET from an observational multicenter cohort, the German Stroke Registry – Endovascular Treatment trial. Procedural characteristics and functional outcome at discharge and 90 days were compared between patients with and without ongoing IVT at flow restoration. To determine associations with functional outcome, adjusted ORs were calculated using ordinal multivariable logistic regression models adjusted for potential baseline confounder variables.

**Results:**

Among 1303 patients treated with IVT and ET who achieved successful recanalization, IVT was ongoing in 13.8% (n=180) at flow restoration. Ongoing IVT was associated with better functional outcome at discharge (adjusted OR 1.61; 95% CI 1.13 to 2.30) and at 90 days (adjusted OR 1.52; 95% CI 1.06 to 2.18).

**Conclusion:**

These results provide preliminary evidence for a benefit of ongoing IVT at flow restoration in patients with AIS treated with ET.

**What is already known on this topic**

- Endovascular thrombectomy and IV thrombolysis are standard of care in eligible patients with large vessel occlusion stroke. The CHOICE trial reported improved outcome in patients receiving additional IA thrombolysis after successful thrombectomy. We hypothesized that ongoing IV thrombolysis on flow restoration might show a similar benefit, but this has not been investigated to date.

**What this study adds**

- Ongoing IV thrombolysis on flow restoration might be associated with improved clinical outcome in large vessel occlusion stroke.

**How this study might affect research, practice or policy**

- Alternative thrombolysis regimes such as an application over a longer time interval, deferred application of a partial dose on flow restoration or thrombolytics with extended serum half-life might exploit the observed effect and warrant clinical investigation.

## Introduction

Endovascular thrombectomy (ET) and intravenous thrombolysis (IVT) are the optimal treatment in eligible patients with large vessel occlusion (LVO) acute ischemic stroke (AIS).^1^ Flow restoration is evaluated using the modified Treatment in Cerebral Ischemia (mTICI) or expanded TICI (eTICI) score.^2^ Successful recanalization is usually defined as a reperfusion of more than 50% of the initially occluded arterial territory, corresponding to an mTICI score of ≥2b or eTICI score of ≥2b50. However, only 46% of patients achieved good functional outcome at 90 days despite successful recanalization in 71% of patients in randomized trials,^3^ a discrepancy attributed to impaired microcirculatory reperfusion despite successful angiographic recanalization.^4^ In support of this notion, the randomized CHOICE trial, which investigated the benefit of local IA thrombolysis following successful recanalization, found a greater likelihood of excellent clinical outcome at 90 days, defined as a modified Rankin Scale score (mRS) of 0 or 1.^5^ This benefit might result from more effective thrombolytic activity within the microcirculation after removal of more proximal occlusions, improving microcirculatory reperfusion.^5 6^ We therefore tested the hypothesis that ongoing systemic IVT on successful recanalization is associated with improved outcome in patients with AIS treated with ET in a large multicenter cohort.

## Methods

The German Stroke Registry – Endovascular Treatment (GSR-ET; NCT03356392) is a prospective multicenter registry of patients with AIS with LVO treated by ET. Patients were recruited between June 2015 and April 2018 from 25 hospitals in Germany. Details have been published previously.^7^ The study was conducted in accordance with the Declaration of Helsinki and was centrally approved by the institutional review board of the Ludwig-Maximilians University Munich (689-15) and institutional review boards according to local regulations.

GSR-ET patients were included in this analysis according to the following inclusion criteria: (1) treatment with IVT; (2) successful angiographic recanalization (mTICI ≥2b); and (3) documented times of symptom onset, IVT and flow restoration. Ongoing IVT was assumed if flow restoration occurred within 60 min after initiation of IVT. The neurological endpoint was functional outcome as measured by mRS at discharge and 90 days. Post-interventional symptomatic intracranial hemorrhage (ICH) was graded according to the European Cooperative Acute Stroke Study criteria.^8^

Baseline characteristics, complications and procedural results were compared using Fisher’s exact test, Mann–Whitney U test or unpaired Student’s t-test. To compare outcome between patients with and without ongoing IVT on flow restoration, we performed ordinal multivariable logistic regression adjusting for pre-specified potential baseline confounder variables (age, sex, National Institutes of Health Stroke Scale (NIHSS) score on admission, baseline Alberta Stroke Program Early CT Score (ASPECTS), premorbid mRS, time from symptom onset to flow restoration, number of thrombectomy maneuvers and diabetes). The significance level was α=0.05. All analyses were performed with R version 4.0.3 (R Core Team, 2020).

## Results

A total of 1303 patients with AIS with successful flow restoration following IVT and ET were eligible for the study, 180 (13.8%) of whom had ongoing IVT on flow restoration (online supplemental figure 1). Patients with ongoing IVT at flow restoration had a higher baseline ASPECTS, were less frequently referred for ET, and required fewer retrieval attempts. Furthermore, the interval from symptom onset to flow restoration was shorter, while the interval from symptom onset to IVT was similar (table 1).

10.1136/jnis-2022-019522.supp1 Supplementary data

**Table 1:** Baseline characteristics and periprocedural results

	IVT ongoing at FLR n=180	IVT finished at FLR n=1123	P value
Age (years), mean±SD	71.8±14.5	71.9±13.4	0.94
Female sex, % (n)	48.3 (87)	48.1 (540)	1.0
Prestroke mRS, median (IQR)	0 (0–0)	0 (0–1)	0.74
NIHSS, median (IQR)	14 (7–18)	14 (9–18)	0.06
ASPECTS, median (IQR)	10 (8–10)	9 (8-10)	0.015
Cardiovascular risk factors, % (n)			
Hypertension	41.7 (75)	38.5 (430)	0.46
Diabetes	70.9 (127)	73.0 (815)	0.59
Dyslipidemia	15.6 (28)	20.8 (232)	0.11
Atrial fibrillation	40.0 (72)	33.9 (379)	0.13
Current smoking	15.8 (27)	16.7 (170)	0.83
Referral for ET, % (n)	7.8 (14)	46.0 (517)	<0.001
Thrombectomy maneuvers (IQR)	1 (1–2)	2 (1–3)	<0.001
Time intervals (min), median (IQR)			
Symptom onset to IVT	92.5 (80-135)	90 (70–125)	0.055
Symptom onset to groin	117 (100–149)	180.5 (140-240)	<0.001
IVT to FLR	47 (39–53)	128 (91–180)	<0.001
Groin to FLR	22 (15–30)	41 (26–61)	<0.001
Symptom onset to FLR	141 (121–176)	229 (185–290)	<0.001
Complications, % (n)			
Device malfunction	0.6 (1)	0.1 (1)	0.26
Dissection or perforation	1.7 (3)	1.8 (20)	1.0
ICH after 24 hours	6.1 (11)	14.8 (166)	0.001
Symptomatic ICH	1.1 (2)	3.9 (43)	0.08

ASPECTS, Alberta Stroke Program Early CT Score; FLR, flow restoration; ICH, intracranial hemorrhage; IVT, intravenous thrombolysis; mRS, modified Rankin Scale; NIHSS, National Institutes of Health Stroke Scale.

**Figure 1:**
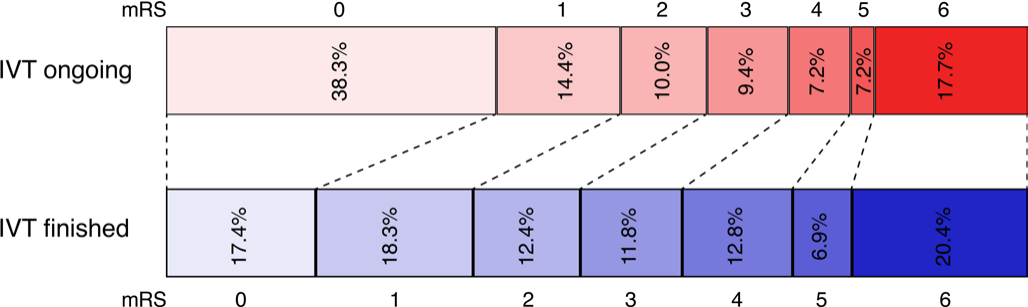
Functional outcome in patients with acute ischemic stroke treated with thrombectomy and IV thrombolysis (IVT) with and without ongoing IVT at flow restoration. Distribution of modified Rankin Scale (mRS) scores at 90 days follow-up.

An excellent clinical outcome (mRS 0 or 1) at 90 days was achieved in 52.8% of patients with ongoing IVT at flow restoration compared with 36.0% of patients without ongoing IVT (figure 1, p<0.001). Peri-procedural complications were similar in both groups except for a lower frequency of any ICH reported at 24 hours in patients with ongoing IVT at flow restoration (table 1).

After adjustment for potential confounders, ongoing IVT at flow restoration was associated with better functional outcome at discharge (adjusted OR 1.61; 95% CI 1.13 to 2.30) and at 90 days (adjusted OR 1.52; 95% CI 1.06 to 2.18, table 2).

**Table 2:** Ongoing IVT on flow restoration predicts functional outcome in multivariable analysis

	mRS at discharge			mRS at 90 days		
	aOR	95% CI	P value	aOR	95% CI	P value
Age	0.96	0.95 to 0.97	<0.001	0.95	0.94 to 0.96	<0.001
Female sex	1.22	0.96 to 1.54	0.10	0.94	0.74 to 1.19	0.62
NIHSS	0.89	0.87 to 0.91	<0.001	0.90	0.88 to 0.92	<0.001
ASPECTS	1.25	1.16 to 1.35	<0.001	1.23	1.14 to 1.33	<0.001
Diabetes	0.46	0.35 to 0.61	<0.001	0.44	0.33 to 0.59	<0.001
SO to flow restoration, hours	0.85	0.79 to 0.92	<0.001	0.84	0.77 to 0.91	<0.001
Premorbid mRS	0.65	0.58 to 0.73	<0.001	0.64	0.57 to 0.71	<0.001
Ongoing IVT	1.61	1.13 to 2.30	0.009	1.52	1.06 to 2.18	0.025
Retrieval attempts	0.77	0.71 to 0.84	<0.001	0.82	0.75 to 0.89	<0.001

aOR, adjusted OR; ASPECTS, Alberta Stroke Program Early CT Score; IVT, intravenous thrombolysis; mRS, modified Rankin Scale; NIHSS, National Institutes of Health Stroke Scale; SO, symptom onset.

## Discussion

Based on real-world data from 1303 patients with LVO achieving successful flow restoration following treatment with ET and IVT, our study suggests a better functional outcome in patients with ongoing IVT at flow restoration.

Our findings confirm the results of the CHOICE trial, which reported increased rates of excellent clinical outcome in patients receiving local IA thrombolysis after recanalization (59.0% vs 40.4%). The shift analysis did not reach statistical significance (OR 1.54, 95% CI 0.79 to 2.94),^5^ but premature termination of the trial reduced its statistical power. Of note, IA thrombolysis was applied irrespectively of systemic thrombolysis, which was administered in 57% of patients. Although reported intracranial bleeding rates were low and no clear signal was found in safety analyses, these data have to be confirmed in larger analyses before implementation in clinical routine.^5^

The frequency of symptomatic ICH in our cohort was similar to recent data.^3 9^ Although not statistically significant, symptomatic ICH was less frequent in patients with ongoing IVT at flow restoration, which agrees with the promising safety signal observed in the CHOICE trial.^5^

The strengths of our study include the large sample size and use of prospectively collected data from a nationwide registry. A limitation of the study is its observational character, and residual confounding cannot be excluded. Furthermore, there was no central assessment of successful flow restoration by an independent core laboratory, and the subgroup allocation of ongoing versus finished IVT at flow restoration was retrospectively derived from reported time metrics. Most importantly, major prognostic factors were imbalanced between the two groups, which, despite multivariable analysis, might account for the observed outcome differences to some extent.

If further confirmed, the preliminary evidence provided in our study for improved clinical outcome in patients with LVO receiving IVT at flow restoration would have several implications. Pre-ET application of IVT is clearly associated with improved outcome, and delaying IVT initiation to coincide with flow restoration will likely be disadvantageous.^10^ Achieving flow restoration within 60 min is neither plannable nor feasible in the majority of cases. However, IVT application over a longer time interval, deferred application of a partial dose during flow restoration, or using a recombinant tissue-type plasminogen activator with a longer serum half-life such as tenecteplase are among possible approaches to improve outcome after successful flow restoration. Indeed, the most pronounced benefit of tenecteplase over alteplase in the recently published AcT trial occurred in the subgroup of patients with LVO stroke,^9^ as the longer serum half life of tenecteplase might convey a similar beneficial effect after flow restoration to IA thrombolysis or ongoing alteplase administration.

10.1136/jnis-2022-019522.supp2 Supplementary data

## Data Availability

The data supporting the findings of this study are available from the corresponding author on reasonable request.
