# Supplementary material for: Intravenous thrombolysis upon flow restoration improves outcome in endovascular thrombectomy
Source: J Neurointerv Surg. 2022 Oct 28;15(e2):e229–31. doi: 10.1136/jnis-2022-019522 (PMC10646906; doi:10.1136/jnis-2022-019522)
Supplement: Supplementary data [file jnis-2022-019522supp001.pdf]

BMJ Publishing Group Limited (BMJ) disclaims all liability and responsibility arising from any reliance placed on this supplemental material which has been supplied by the author(s)


# SUPPLEMENTAL MATERIAL

## Intravenous thrombolysis upon flow restoration improves outcome in endovascular thrombectomy

**Supplementary Figure 1.** Flow diagram for patient inclusion.

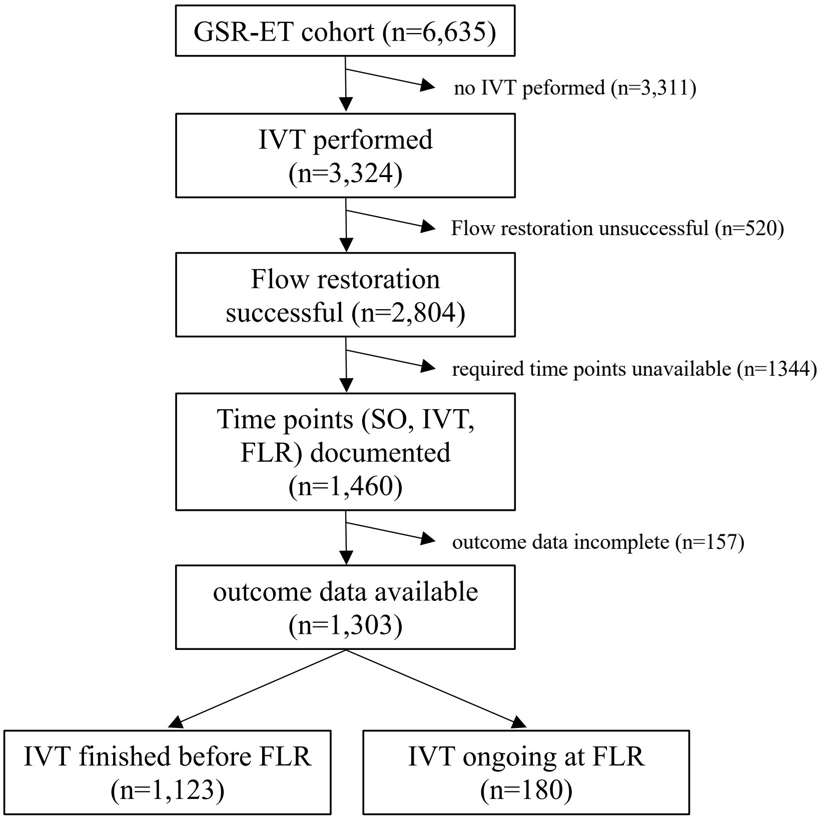


*SO indicates symptom onset; IVT, intravenous thrombolysis; FLR, flow restoration.*

**GSR-ET Collaborators**

A. Alegiani, J. Berrouschot, T. Boeck-Behrens, G. Bohner, J. Borggrefe, A. Bormann, M. Braun, B. Eckert, R.M. Eckert, U. Ernemann, M. Ernst, J. Fiehler, C. Gerloff, K. Gröschel, G.F. Hamann, K.H. Henn, L. Kellert, C. Kraemer, H. Leischner, J. Liman, A. Ludolph, O. Nikoubashman, C.H. Nolte, M. Petersen, S. Poli, A. Reich, J. Röther, J.H. Schäfer, P. Schellinger, E. Siebert, F. Stögbauer, G. Thomalla, S. Tiedt, C. Trumm, T. Uphaus, S. Wunderlich.
